# Integrating Social and Clinical Determinants of Pre‐Eclampsia: A Hierarchical Systematic Review and Conceptual Framework for Prevention

**DOI:** 10.1111/1471-0528.70240

**Published:** 2026-04-14

**Authors:** Mai‐Lei Woo Kinshella, Jeffrey N. Bone, Terteel Elawad, Hiten D. Mistry, Laney Poye, Eleni Tsigas, Deepika Devadas, Violet Mateljan, Marie‐Laure Volvert, Marianne Vidler, Hannah Blencowe, Véronique Filippi, Laura A. Magee, Peter von Dadelszen, The PRECISE Network, Umberto D'Alessandro, Anna Roca, Hawanatu Jah, Andrew Prentice, Melisa Martinez‐Alvarez, Brahima Diallo, Abdul Sesay, Sambou Suso, Baboucarr Njie, Fatima Touray, Yahaya Idris, Fatoumata Kongira, Modou F. S. Ndure, Lawrence Gibba, Abdoulie Bah, Yorro Bah, Marleen Temmerman, Angela Koech, Patricia Okiro, Geoffrey Omuse, Grace Mwashigadi, Mary Goretti Amondi, Consolata Juma, Joseph Mutunga, Moses Mukhanya, Robin Okello, Onesmus Wanje, Isaac Mwaniki, Marvin Ochieng, Emily Mwadime, Esperança Sevene, Corssino Tchavana, Salesio Macuacua, Anifa Vala, Helena Boene, Lazaro Quimice, Sonia Maculuve, Inacio Mandomando, Peter von Dadelszen, Laura A. Magee, Rachel Craik, Marie‐Laure Volvert, Hiten Mistry, Thomas Mendy, Donna Russell, Prestige Tatenda Makanga, Liberty Makacha, Reason Mlambo, Lucilla Poston, Rachel Tribe, Sophie Moore, Tatiana Salisbury, Aris Papageorghiou, Alison Noble, Rachel Craik, Hannah Blencowe, Veronique Filippi, Joy Lawn, Matt Silver, Joseph Akuze, Ursula Gazeley, Judith Cartwright, Guy Whitley, Sanjeev Krishna, Marianne Vidler, Jing (Larry) Li, Jeff Bone, Mai‐Lei (Maggie) Woo Kinshella, Domena Tu, Ash Sandhu, Kelly Pickerill, Carla Carillho, Benjamin Barratt

**Affiliations:** ^1^ Department of Women and Children's Health, School of Life Course and Population Sciences Kings College London London UK; ^2^ Department of Obstetrics and Gynaecology BC Children's and Women's Hospital and University of British Columbia Vancouver Canada; ^3^ BC Children's Hospital Research Institute Vancouver British Columbia Canada; ^4^ Royal Free London NHS Foundation Trust London UK; ^5^ Division of Public Health and Epidemiology, College of Life Sciences University of Leicester Leicester UK; ^6^ Preeclampsia Foundation Melbourne Florida USA; ^7^ Preeclampsia Foundation Canada Fonthill Ontario Canada; ^8^ Faculty of Epidemiology and Population Health London School of Hygiene & Tropical Medicine London UK

**Keywords:** conceptual framework, determinants of health, pre‐eclampsia, social and clinical

## Abstract

**Background:**

Pre‐eclampsia is a leading cause of maternal and perinatal morbidity and mortality, with risk factors reported across a vast literature base fragmented between social and clinical factors.

**Objective:**

To develop a comprehensive conceptual framework of the strongest risk factors and their relationships contributing to pre‐eclampsia incidence.

**Search Strategy:**

Medline, Embase, Health Technology Assessments and Database of Abstracts of Reviews of Effects, Cochrane Library were searched.

**Selection Criteria:**

Reviews, randomized controlled trials and cohort studies (> 1000 participants), reporting social and clinical factors associated with pre‐eclampsia were included.

**Data Collection and Analysis:**

The strongest factors, defined as those with at least moderate strength of association and quality of evidence using GRADE, were compiled from our previously published individual frameworks to create a combined conceptual framework. Indirect associations were searched and the strongest indirect factors were added.

**Main Results:**

The conceptual framework integrated 35 social and clinical determinants of pre‐eclampsia. Key modifiable factors included BMI, interlinked with chronic hypertension/elevated blood pressure in early pregnancy, type 2 diabetes mellitus, and obstructive sleep apnoea, as well as antenatal care attendance, interconnected with maternal/work stress and prenatal micronutrient supplementation. Other modifiable factors included smoking, antiphospholipid syndrome, infection, exposure to occupational hazards, distance to health facility, maternal heat exposure in early gestation, and UV‐B exposure.

**Conclusion:**

There are strong social factors alongside clinical factors associated with pre‐eclampsia incidence. Interwoven relationships between factors highlight the multifactorial aetiology of pre‐eclampsia. Many determinants were potentially modifiable, which provides actionable intervention points for clinical care and public health strategies.

## 1 Introduction

Pre‐eclampsia is the most serious of the hypertensive disorders of pregnancy (HDP), characterized by *de novo* hypertension at or after 20 weeks gestation alongside proteinuria, maternal end‐organ involvement, and/or uteroplacental dysfunction [1]. Pre‐eclampsia is a leading cause of preventable maternal deaths, associated with over 46 000 maternal and 500 000 perinatal deaths annually [1]. The risk of experiencing severe maternal morbidity is five times higher among women with pre‐eclampsia, compared to normotensive women [2]. Additionally, women with pre‐eclampsia have a higher risk of cardiovascular disease, diabetes, dyslipidaemia, and chronic kidney disease in later life, and their offspring have a higher risk of preterm birth, attention deficit/hyperactivity disorder, increased body mass index (BMI), and cardiovascular disease [3, 4, 5, 6, 7]. Incidence rates of pre‐eclampsia are increasing [8, 9, 10, 11] with global HDP incidence increasing by 15.2% between 1990 and 2021 from 31.3 million to 36.1 million cases [12].

Correspondingly, the number of publications on pre‐eclampsia has substantially increased in recent years, with varying degrees of quality [13]. The growing body of pre‐eclampsia research increasingly acknowledges its complexity and multifactorial aetiology, with multiple pathways of risk in the development of the disease [14, 15, 16]. Inadequate placentation and/or increased fetoplacental demand such as with multiple gestation or macrosomia lead to uteroplacental mismatch, heightened oxidative stress, and the release of syncytiotrophoblast stress–derived factors and imbalanced placental angiogenic factors. These interact with underlying maternal risk factors and promote systemic endothelial dysregulation and inflammation, leading to hypertension in the maternal syndrome [1, 14, 15, 16].

Risk factors for pre‐eclampsia are reported across a vast literature base and an evaluation of clinical practice guidelines found that many reported risk factors were not well aligned with current evidence [17]. Evidence is often fragmented by discipline, which hampers understanding of the complex interplay of risk factors in the development of pre‐eclampsia. Though social factors may influence the development of pre‐eclampsia, these have not been well incorporated into previous reviews of pre‐eclampsia risk factors [18, 19, 20, 21]. As part of the PRECISE (Pregnancy Care Integrating translational Science, Everywhere) Network [22], we sought to develop a comprehensive conceptual framework of the strongest social and clinical risk factors and their relationships contributing to pre‐eclampsia incidence.

## 2 Methods

### 2.1 The PRECISE Network

The PRECISE Network was established to understand the complex pathways to placental disorders in sub‐Saharan Africa, including HDPs, preterm birth, fetal growth restriction, and stillbirth [23]. A prospective cohort of 6932 pregnant women and 1825 non‐pregnant women of reproductive age were recruited in The Gambia (West Africa), Kenya (East Africa), and Mozambique (Southern Africa) [22]. The PRECISE Network consortium (Table S1) undertook the development of a comprehensive conceptual framework for pre‐eclampsia to understand the evidence landscape and inform analyses. We employed a hierarchical systematic review method plus a targeted search for indirect associations; each phase of the review process is further detailed in subsequent sections.

### 2.2 Literature Searches and Data Extraction for Individual Social and Clinical Frameworks

The development of a standardized approach to assess the strength of association and quality of evidence linking social, clinical, and biomarker risk factors for placental disorders has been previously described [24], but is summarized briefly below.

We adapted the methods of Hiatt et al. to develop a criteria‐based model of determinants using a systematic process [24, 25]. The PRECISE Network consortium first developed a broad working model of known determinants of pre‐eclampsia based on umbrella reviews of systematic reviews [19, 20] and prominent social determinants of health frameworks (Healthy People 2030) [26], the World Health Organization [27], Public Health Agency of Canada [28], and the Dahlgren‐Whitehead Rainbow model [29]. The process was also guided by engagement with patient‐partners [24].

The approach was first applied to the development of individual frameworks for clinical factors [17] and social factors including socioeconomic status, social support/exclusion, healthcare access, and occupational and physical environmental factors [30], as well as diet and nutritional factors [31]. Additionally, biomarkers of the development of pre‐eclampsia were also compiled as measurable indicators of underlying biological states and processes [32]. Systematic searches were conducted up to 11 June 2025 using Medline, Embase, Evidence‐Based Medicine (EBM) Reviews, Google Scholar, and reference lists, then a hierarchical approach was utilized to identify the highest level of evidence. EBM Reviews includes the following databases: Health Technology Assessments, Database of Abstracts of Reviews of Effects, and Cochrane Library. Direct associations with pre‐eclampsia in each domain was investigated independently.

We employed a hierarchical approach to first prioritize umbrella reviews (systematic reviews of reviews), followed by systematic reviews with meta‐analyses, randomized controlled trials (RCTs), and lastly, large (at least 1000 participants) observational cohort studies. Following the methodology of Bartsch et al., cohort studies with at least 1000 participants were targeted to be more representative of the general population and to have sufficient statistical power to assess less prevalent risk factors [21]. Other study designs and reports with incomplete methodological data, such as letters to the editor, were excluded. Titles and abstracts of articles were screened against the eligibility criteria, with all potentially eligible studies reviewed in full text independently by at least two members of the review team. Disagreements were discussed with the reviewer team until resolved. Studies were included if they met study design criteria as described above and if they reported quantitative associations between exposure factors and the incidence of pre‐eclampsia. General study characteristics, characteristics to assess quality, and the association with pre‐eclampsia, expressed as odds ratios (OR), relative risk (RR), and hazard ratios (HR), were extracted. All associations with pre‐eclampsia were reported.

### 2.3 Assessing the Strength and Quality of Associations

The strength of association with pre‐eclampsia was evaluated based on criteria adapted from Hiatt et al. [25]. The strength of the relationship was categorized as definite (≥ 3.00 or < 0.33), probable (1.50–2.99 or 0.33–0.67), possible (1.10–1.49 or 0.68–0.89), and unlikely (0.90–1.09). RR and OR were employed interchangeably for the model, as ORs provide a reasonable estimate of the RR when the outcome is observed in less than 10% of both exposed and unexposed populations, which is typical for pre‐eclampsia [33].

The Grading of Recommendations, Assessment, Development, and Evaluation (GRADE) approach was used to assess high, moderate, low, and very low quality of evidence [34, 35]. Following GRADE procedures, reviews and RCTs started as high quality, which could be downgraded due to potential bias (risk of bias assessments in reviews, evidence of sensitivity analyses and adjusting for potential confounders, study limitations), inconsistency (significant variability *I* ^2^ > 50%), imprecise measurements (wide confidence intervals), and potential publication bias (asymmetric funnel plot). Single observational studies were considered low quality evidence that could be upgraded with evidence of a dose response relationship or large effect sizes, as well as downgraded for potential bias, inconsistency, imprecision, and publication bias [35].

### 2.4 Compiling the Combined Framework and Indirect Associations

The strongest factors, defined as those with at least moderate strength of association and quality of evidence, were compiled from the published individual clinical and social frameworks [17, 30, 31], to create a combined framework. Indirect associations (i.e., possibly mediated pathways) between these factors were investigated to map the inter‐relationships, which allows assessment of how the stronger risk factors may associate with pre‐eclampsia. Indirect associations between strong clinical to clinical factors, social to social factors, and clinical to social factors were broadly searched pragmatically across databases to ascertain if any relationship has been established in the literature. Because associations between risk factors may occur outside of pregnancy (i.e., obesity and type 2 diabetes mellitus), eligibility was expanded to women in general where applicable. Indirect associations were then assessed by their strength of association and quality of evidence using GRADE, and those with definite and probable strength of association and moderate‐ to high‐quality evidence were added to the framework. The combined framework also distinguished modifiability, defined as any determinant that could be modified through interventions at either individual or population level. These could include altering societal norms, personal behaviours, and/or clinical pathways. Modifiability for each determinant was assessed by expert consensus by the reviewer team. The final conceptual framework was mapped to illustrate the key factors and their interconnections that disrupt normal pregnancy physiology and care pathways, leading to the development of pre‐eclampsia.

## 3 Results

There were overall 35 strong determinants of pre‐eclampsia identified in the literature that underpins the conceptual framework, based on 22 studies included in the review. These included 14 clinical factors directly associated with pre‐eclampsia and 9 indirect relationships between strong clinical factors, as well as 10 social factors directly associated with pre‐eclampsia and two indirect relationships between strong social factors (Table 1). Of the 35 determinants identified, 31 were considered modifiable.

**TABLE 1:** Definite/probable factors associated with pre‐eclampsia, based on high/moderate quality evidence.

Factors	Direction of association	Strength of association^a^	Quality of evidence^b^
*Clinical determinants for pre‐eclampsia*			
High BMI (kg/m^2^)			
Obesity (BMI ≥ 30)	Risk factor	Definite	High
Overweight (BMI = 25.0–29.9)	Risk factor	Probable	High
Type 2 diabetes mellitus	Risk factor	Definite	Moderate
Obstructive sleep apnoea	Risk factor	Probable	Moderate
High blood pressure			
Chronic hypertension	Risk factor	Definite	Moderate
Booking stage 1 hypertension (sBP ≥ 130 or dBP ≥ 80 mmHg)	Risk factor	Probable	High
Booking elevated blood pressure (sBP 120–129 with dBP < 80 mmHg)	Risk factor	Probable	High
Antiphospholipid syndrome	Risk factor	Probable	Moderate
Prior pre‐eclampsia	Risk factor	Definite	Moderate
Prior stillbirth	Risk factor	Probable	Moderate
Family history of pre‐eclampsia in mother or sister	Risk factor	Probable	Moderate
Fetal trisomy 13	Risk factor	Definite	Moderate
Smoking	Inverse association	Probable	Moderate
Any infection (in current pregnancy)	Risk factor	Probable	Moderate
*Indirect clinical factors*			
Obesity ➔ type 2 diabetes mellitus	Risk factor	Definite	High
Overweight ➔ type 2 diabetes mellitus	Risk factor	Definite	High
Obesity ➔ obstructive sleep apnoea	Risk factor	Definite	High
Overweight ➔ obstructive sleep apnoea	Risk factor	Definite	High
Obesity ➔ hypertension	Risk factor	Definite	High
Overweight ➔ hypertension	Risk factor	Probable	Moderate
Smoking ➔ obesity	Inverse association	Probable	Moderate
Smoking ➔ overweight	Inverse association	Probable	Moderate
OSA ➔ type 2 diabetes mellitus	Risk factor	Probable	Moderate
*Direct social determinants of pre‐eclampsia*			
Work stress	Risk factor	Probable	High
Occupational whole‐body vibrations	Risk factor	Probable	Moderate
Occupational prolonged bending	Risk factor	Probable	Moderate
Lack of ANC	Risk factor	Probable	High
Distance to health facility	Risk factor	Probable	Moderate
Heat exposure in early pregnancy	Risk factor	Probable	High
UV‐B exposure	Protective factor	Probable	Moderate
Micronutrient supplementation			
Calcium	Protective factor	Probable	Moderate
Vitamin D and calcium	Protective factor	Probable	High
Vitamin D	Protective factor	Probable	High
*Indirect social factors*			
Maternal stress ➔ fewer ANC visits	Risk factor	Probable	Moderate
High ANC attendance ➔ calcium supplementation	Protective factor	Definite	High

Abbreviations: ANC, antenatal care; dBP, diastolic blood pressure; sBP, systolic blood pressure; UV‐B, ultraviolet B.

^a^ Strength of association was assessed as definite when odds ratios/relative risk was ≥ 3.00 or < 0.33 (presented in dark green boxes) and probable for 1.50–2.99 or 0.33–0.67 (in light green).

^b^ Quality of evidence was assessed as high (in dark orange) and moderate (in light orange) according to GRADE.

### 3.1 Direct and Indirect Clinical Determinants of Pre‐Eclampsia

Clinical determinants for pre‐eclampsia with definite and probable strength of association, based on moderate‐ to high‐quality evidence, included high BMI (obesity [BMI ≥ 30] and overweight [BMI = 25.0–29.9]), type 2 diabetes mellitus, obstructive sleep apnoea, high blood pressure (chronic hypertension, stage 1 hypertension [systolic ≥ 130 or diastolic ≥ 80 mmHg] and elevated blood pressure [systolic 120–129 with diastolic < 80 mmHg] at booking), antiphospholipid syndrome, prior stillbirth, prior pre‐eclampsia, family history of pre‐eclampsia in mother or sister, fetal trisomy 13, smoking and any infection in current pregnancy [17] (Table 1). All were risk factors except smoking, which had an inverse association with pre‐eclampsia based on moderate quality evidence.

Between clinical factors associated with pre‐eclampsia, indirect relationships with at least probable strength of association and moderate quality evidence included associations between high BMI and type 2 diabetes [36], obstructive sleep apnoea [37], and hypertension [36], and obstructive sleep apnoea with type 2 diabetes [38] (Table 1). Smoking had an inverse association with obesity and overweight [39], based on moderate‐quality evidence. Other potential associations included high BMI and stage 1 hypertension [40], and diabetes mellitus with high blood pressure [41], based on low‐ to very low‐quality evidence (Table S2). Smoking was a possible risk factor for type 2 diabetes [42], obstructive sleep apnoea [43] and chronic hypertension [44] based on moderate‐ to very low‐quality evidence.

### 3.2 Direct and Indirect Social Determinants of Pre‐Eclampsia

Social determinants for pre‐eclampsia with definite and probable strength of association, based on high to moderate quality evidence, included work stress, occupational whole‐body vibration and prolonged bending, lack of antenatal care (ANC), distance to health facility, heat exposure in early pregnancy, UV‐B exposure, and micronutrient supplementation (calcium and/or vitamin D supplementation) [30, 31](Table 1). UV‐B exposure and calcium and/or vitamin D supplementation were protective factors; all others were risk factors.

Between social factors associated with pre‐eclampsia, indirect relationships with at least probable strength of association and moderate quality evidence included associations between maternal stress and fewer ANC visits [45], and between high ANC attendance and increased calcium supplementation [46] (Table 1). Other potential indirect associations included heat exposure on maternal stress [47], and distance to health facility on delayed ANC [48], based on low‐quality evidence (Table S3).

### 3.3 Indirect Relationships Between Clinical and Social Determinants of Pre‐Eclampsia

No associations with definite or probable strength, based on high‐ to moderate‐quality evidence, were found. Possible associations included work stress and type 2 diabetes [49]—based on high‐quality evidence, UV‐B exposure and hypertension [50]—based on moderate‐quality evidence, and work stress and higher BMI [51], work stress and smoking in pregnancy [52], smoking with fewer ANC visits [53], and overweight with late ANC booking [54]—based on low‐quality evidence (Table S4). Systematic reviews on calcium supplementation and calcium, plus vitamin D supplementation did not find any studies in women that reported hypertension as a dichotomous outcome [55, 56], though the Cochrane review found lower diastolic blood pressure among women who received calcium supplementation compared to controls (MD −1.04, 95% CI −1.86, −0.22) [55].

### 3.4 Conceptual Framework

**FIGURE 1:**
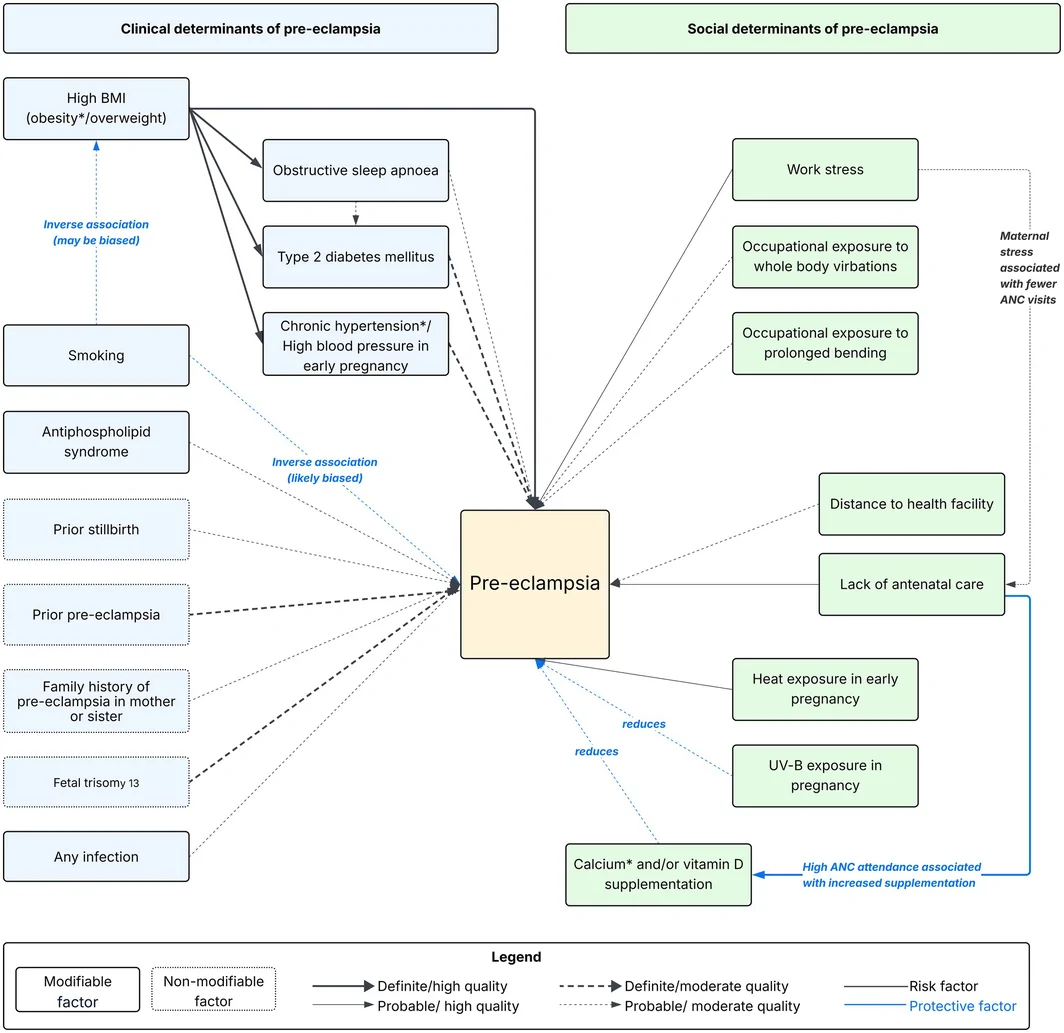
Conceptual framework for pre‐eclampsia prevention, based on definite/probable associations with high/moderate quality evidence. Definite (≥ 3.00 or < 0.33) or probable (1.50–2.99 or 0.33–0.67) strength of association; High or moderate quality of evidence assessed using GRADE. *Indicates the factor illustrated where a node included multiple factors.

A conceptual framework for pre‐eclampsia prevention, based on definite/probable associations with high/moderate quality evidence is illustrated in Figure 1. High BMI, obstructive sleep apnoea, type 2 diabetes, high blood pressure, smoking, antiphospholipid syndrome and any infection were considered modifiable clinical factors. Prior stillbirth, prior pre‐eclampsia, family history of pre‐eclampsia in mother or sister, and fetal trisomy 13 as a genetic condition were considered non‐modifiable factors. All social determinants of pre‐eclampsia were considered modifiable factors. The strongest modifiable clinical pathways were with BMI, either directly or through type 2 diabetes and chronic hypertension/high blood pressure. ANC attendance appeared to be a strong factor within social pathways, as maternal stress may be associated with lower ANC attendance while high ANC attendance appears required for optimal calcium supplementation especially in global resource‐limited settings [46].

## 4 Discussion

### 4.1 Summary of Findings and Comparison to Current Literature

This framework provides, for the first time, an integrated synthesis of social and clinical determinants of pre‐eclampsia based on systematically graded evidence. It advances prior reviews that focused on individual domains by mapping inter‐relationships across metabolic, cardiovascular, environmental, and healthcare pathways.

### 4.2 Integration With the Existing Literature

Our findings reaffirm several well‐established clinical risk factors, including obesity, type 2 diabetes, and chronic hypertension [18, 19, 20, 21], and furthermore highlight their inter‐relationship with each other and obstructive sleep apnoea to increase the risk of pre‐eclampsia incidence. Addressing a key gap in pre‐eclampsia research, this framework is strengthened by the inclusion of social determinants including several occupational risk factors, environmental exposures, and access to quality antenatal care. Early and consistent healthcare engagement during pregnancy provides opportunities for preventative measures, such as the relationship between ANC attendance and calcium and vitamin D supplementation reported in our framework, and supports identifying those with risk factors for early detection and treatment, such as the clinical risk factors described above. Underlying social factors may in turn negatively influence ANC attendance, such as stressful life events and work stress. By incorporating a broad range of direct and indirect associations, this work sheds insight into possible pathways in the development of pre‐eclampsia and provides a way to assess existing data and plan future research. Many determinants identified were considered modifiable, which lends to actionable intervention points for clinical care and public health strategies.

The indirect associations between high BMI, type 2 diabetes and hypertension highlights the metabolic and cardiovascular pathways in the pathophysiology of pre‐eclampsia, especially given concern on the increasing global burden of non‐communicable diseases [57]. These maternal risk factors often combine to increase risk of the development of pre‐eclampsia, particularly late onset pre‐eclampsia (≥ 34 weeks' gestation), which accounts for at least 70% of pre‐eclampsia cases [1, 14, 15, 16, 58]. A study from the United States found that obesity was associated with pre‐eclampsia within the overall cohort (RR 1.41, 95% CI: 1.30–1.53), but the relationship was only significant in sub‐cohorts of other risk factors, especially chronic hypertension, prior pre‐eclampsia, diabetes, kidney disease, and/or autoimmune conditions [59]. This supports our findings on indirect associations and underscores the importance of clinical care strategies to manage chronic hypertension during pregnancy, particularly among women with high BMI. The CHIPS (Control of Hypertension In Pregnancy Study) and CHAP (Chronic Hypertension and Pregnancy Trial) trials previously found that the use of antihypertensive therapy to normalize blood pressure during pregnancy was associated with fewer maternal complications, with no increase in adverse perinatal outcomes [60, 61]. Tight glycemic control alongside lifestyle interventions to support appropriate gestational weight gain, low‐dose aspirin and antihypertensive treatment if necessary is recommended for women with type 2 diabetes in pregnancy [62]. It is of note that there is still substantial uncertainty around the effectiveness of type 2 diabetes therapies to reduce the risk of pre‐eclampsia [63, 64]. Findings on indirect associations between pre‐eclampsia risk factors can be used to inform future mediation analyses to further disentangle these etiological relationships, that is, into direct and mediated effects.

### 4.3 Implications for Clinical Care and Public Health

The management of cardiovascular and metabolic risk factors, and infections such as urinary tract infections and periodontal disease [65], emphasizes the crucial access to quality healthcare services during pregnancy. Work stress and other stressful life events were associated with fewer ANC visits, and high ANC attendance may be required for optimal healthcare engagement during pregnancy, such as micronutrient supplementation when needed. An analysis of 276 388 mother‐infant dyads in the World Health Organization Global Survey on Maternal and Perinatal Health found that more than 8 ANC visits were associated with 10% reduced odds of pre‐eclampsia (adjusted OR 0.90, 95% CI: 0.83–0.98) [66]. A Cameroon study found that calcium supplementation was strongly associated with women with more than three ANC visits (adjusted OR 6.01, 95% CI: 3.84–9.34) and early ANC before 4 months of pregnancy (adjusted OR 2.64, 95% CI: 1.84–3.78) [46]. This framework found that calcium and/or vitamin D supplementation is probably protective, though certainty of evidence is challenged by substantial heterogeneity between studies [67] and frequently unaccounted sources such as water for calcium [68] and UV‐B for vitamin D [69]. Low baseline calcium intake may be a determining factor for the effectiveness of calcium supplementation, which reaffirms the need for quality antenatal support in resource‐limited settings where low dietary intakes are more prevalent, and personalized care in high‐income settings to identify women with low dietary intakes [70]. Though prior stillbirth, previous pre‐eclampsia, family history of pre‐eclampsia, and fetal trisomy 13 are not modifiable, improved screening during antenatal care can support pre‐eclampsia prevention strategies and early detection for a better prognosis.

There are two areas of the conceptual framework that require nuanced interpretation. First, the inverse association between smoking and pre‐eclampsia likely reflects methodological artefacts, including left truncation bias, misclassification, and inappropriate adjustment, rather than a true protective effect [71, 72, 73]. Smoking is associated with altered placental development and higher oxidative stress implicated in the pathophysiology of pre‐eclampsia [74]. Secondly, though the incidence of HDPs is often reported to be higher among Black and African‐American women, disparities may be strongly related to experiences of social inequities and a higher rate of clinical co‐morbidities [75, 76, 77, 78]. For example, two cohorts from the United States reported no association between Black race and pre‐eclampsia after adjusting for sociodemographic and cardiovascular risk factors [59, 77].

### 4.4 Implications for Research and Advocacy

The conceptual framework can also be used to assess existing data on pre‐eclampsia risk factors and inform possible impact of unmeasured confounders that can occur when key risk factors are unavailable or unaccounted for in statistical modelling [79]. In turn, compiling the strongest associations across pre‐eclampsia literature can illuminate unrepresented relationships. We did not identify any strong indirect associations between social and clinical domains, which may reflect the current fragmentation of research across disciplines and frequent examination of clinical and social factors in isolation. Several possible links, such as between work stress and BMI [51, 80, 81], work stress and type 2 diabetes [49], high BMI and delayed ANC attendance [54], and smoking with fewer ANC visits [53], warrant further investigation. Future research can also explore if uncontrolled chronic hypertension and/or other co‐morbidities contribute to the relationship between inadequate ANC and pre‐eclampsia. However, the relationships between high BMI and smoking with sub‐optimal ANC emphasize the need for careful consideration of potentially stigmatizing risk factors within clinical care, such as BMI, which is influenced by socio‐economic determinants [82, 83].

For a risk factor to be modifiable, it has been argued that the factor must be measurable, have potential for change, be plausible as a cause for the adverse health outcome of interest or related to an underlying plausible cause [84]. Additionally, modifiability is strengthened if there is empirical evidence of an effect [84]. Our conceptual framework identified several modifiable risk factors supported by quality evidence and the inclusion of indirect associations helps to map pathways. However, while modifiable risk factors are potentially changeable, it is important to highlight the need for advocacy on multiple levels. For example, the associations with obstructive sleep apnoea, chronic hypertension and elevated blood pressure, and type 2 diabetes support acting on a clinical level including advocating for awareness and changes in practice. On the other hand, strengthening access to quality ANC services and occupational protection requires acting on a systemic level and advocating with policy makers. Some factors may be modifiable on multiple levels; for example, diet and exercise interventions can support efforts towards a healthy BMI for individuals, but also require attention to upstream factors, such as improving access to healthy foods, creation of safe and inclusive recreational spaces and active transport infrastructure [85, 86]. The causal effects of these multiple intervention strategies on pre‐eclampsia may vary, and therefore the reported measures in this review cannot be directly translated to expected benefit without strong assumptions. Our comprehensive review and framework will allow for more thoughtful and informed estimation of such causal effects in future studies. Additional future work includes working with patient advocacy groups to develop a list of risk factors based on the determinants of pre‐eclampsia conceptual framework that families can use to springboard discussion with their healthcare providers to support personalized care.

### 4.5 Strengths and Limitations

While the hierarchical review methodology allowed us to screen a broad landscape of literature, our review is limited by the evidence available. Weak or absent associations may be reflective of methodological constraints. For example, the prioritization of reviews could limit the inclusion of emerging risk factors not yet reported in a meta‐analysis. Moreover, factors that have been variably defined across the literature or highly contextual to local settings, such as socio‐economic status, are difficult to pool in meta‐analyses. Restricting to large cohort studies, smaller effect sizes reported in population‐based studies and lower evidence certainty of single observational studies especially disadvantage the inclusion of social determinants in the conceptual framework. Most of the evidence originates from studies in high income settings. Though searches for indirect associations between pre‐eclampsia determinants were broadened beyond pregnancy to women in general where applicable, evidence was challenged by gaps in sex‐based analysis and reporting, which has been previously reported in cardiovascular clinical research [87, 88]. Nevertheless, our conceptual framework for pre‐eclampsia prevention is strengthened by the intentional integration of social and clinical factors, assessment of evidence quality, methodological development with patient partners, and highlighting modifiability in the framework as a critical lens for public health planning and advocacy.

## 5 Conclusion

This conceptual framework integrates 35 social and clinical factors with definite or probable associations and high or moderate quality evidence, underscoring the complex interplay of metabolic, cardiovascular, environmental, and healthcare factors. It highlights modifiable determinants (such as high BMI, hypertension, diabetes, infection, and limited antenatal care) as potential leverage points for prevention and system‐level action. By identifying modifiable and non‐modifiable determinants and their interconnections, this framework provides a foundation for causal modelling and targeted prevention strategies to improve maternal and perinatal outcomes.

## Author Contributions

The initial conceptual framework plan was developed by P.D., L.A.M., M.‐L.V. and V.F. The conceptual framework analyses were performed primarily by T.E., M.‐L.W.K. and J.N.B. The first draft of the manuscript was written by M.‐L.W.K., L.A.M. and P.D. All authors participated in manuscript revisions and approved the submitted version.

## Funding

This work was supported by UK Research and Innovation.

## Conflicts of Interest

The authors declare no conflicts of interest.

## Supporting information


**Appendix S1:** bjo70240‐sup‐0001‐appendix.docx.
**Table S1:** The PRECISE Network.
**Table S2:** Indirect relationships for CLINICAL risk factors for pre‐eclampsia, that are definite/probable in nature, based on high/moderate quality evidence*.
**Table S3:** Indirect relationships for SOCIAL risk factors for pre‐eclampsia, that are definite/probable in nature, based on high/moderate quality evidence*.
**Table S4:** Indirect relationships between CLINICAL and SOCIAL risk factors for pre‐eclampsia.

## Data Availability

Data sharing not applicable to this article as no datasets were generated or analysed during the current study.
