# Supplementary material for: Integrating Social and Clinical Determinants of Pre‐Eclampsia: A Hierarchical Systematic Review and Conceptual Framework for Prevention
Source: BJOG. 2026 Apr 14;133(Suppl 4):S43–53. doi: 10.1111/1471-0528.70240 (PMC13572890; doi:10.1111/1471-0528.70240)
Supplement: Supplementary file 1 [file BJO-133-S43-s001.docx]

**Supplementary appendix**

|  | Title | Page number |
| --- | --- | --- |
| **Tables** |  |  |
| Table S1 | The PRECISE Network | 1 |
| Table S2 | Indirect relationships for CLINICAL risk factors for pre-eclampsia, that are definite/probable in nature, based on high/moderate quality evidence | 3 |
| Table S3 | Indirect relationships for SOCIAL risk factors for pre-eclampsia, that are definite/probable in nature, based on high/moderate quality evidence | 9 |
| Table S4 | Indirect relationships between CLINICAL and SOCIAL risk factors for pre-eclampsia | 11 |

**Table S1**: The PRECISE Network

| **In-country teams** | **Members** |
| --- | --- |
| THE GAMBIA: Medical Research Council Unit The Gambia at the London School of Hygiene and Tropical Medicine, Fajara | Umberto D’Alessandro, Anna Roca, Hawanatu Jah, Andrew Prentice, Melisa Martinez-Alvarez, Brahima Diallo, Abdul Sesay, Sambou Suso, Baboucarr Njie, Fatima Touray, Yahaya Idris, Fatoumata Kongira, Modou F.S. Ndure, Lawrence Gibba, Abdoulie Bah and Yorro Bah. |
| KENYA: Aga Khan University, Nairobi | Marleen Temmerman, Angela Koech, Patricia Okiro, Geoffrey Omuse, Grace Mwashigadi, Mary Goretti Amondi, Consolata Juma, Joseph Mutunga, Moses Mukhanya, Robin Okello, Onesmus Wanje, Isaac Mwaniki, Marvin Ochieng, Emily Mwadime |
| MOZAMBIQUE : Centro de Investigação em Saúde de Manhiça, Manhiça | Esperança Sevene, Corssino Tchavana, Salesio Macuacua, Anifa Vala, Helena Boene, Lazaro Quimice, Sonia Maculuve, Inacio Mandomando |
| **Central co-ordinating team** |  |
| Department of Women and Children’s Health, School of Life Course Sciences, Faculty of Life Sciences and Medicine, King’s College London | Peter von Dadelszen, Laura A. Magee, Rachel Craik, Marie-Laure Volvert, Hiten Mistry, Thomas Mendy |
| Donna Russell Consulting | Donna Russell |
| **Co-Investigator team** |  |
| Midlands State University, Zimbabwe | Prestige Tatenda Makanga, Liberty Makacha and Reason Mlambo |
| Kings College London | Lucilla Poston, Rachel Tribe, Sophie Moore, Tatiana Salisbury |
| University of Oxford | Aris Papageorghiou, Alison Noble, Rachel Craik |
| London School of Hygiene and Tropical Medicine | Hannah Blencowe, Veronique Filippi, Joy Lawn, Matt Silver, Joseph Akuze and Ursula Gazeley |
| St George’s, University of London | Judith Cartwright, Guy Whitley, Sanjeev Krishna |
| University of British Columbia | Marianne Vidler, Jing (Larry) Li, Jeff Bone, Mai-Lei (Maggie) Woo Kinshella, Domena Tu, Ash Sandhu, Kelly Pickerill |
| Eduardo Mondlane University, Maputo | Carla Carillho |
| Imperial College London | Benjamin Barratt |

**Table S2**: Indirect relationships for CLINICAL risk factors for pre-eclampsia, that are definite/probable in nature, based on high/moderate quality evidence*

|  | **BMI** | |  |  |  |  | **Blood pressure** | | |  |  |  |  |  |  |  |
| --- | --- | --- | --- | --- | --- | --- | --- | --- | --- | --- | --- | --- | --- | --- | --- | --- |
|  | Obesity | Overwt |  | T2DM | OSA |  | cHTN | Booking Stage 1 HTN | Booking ‘elevated BP’ |  | APAS | Prior PETǂ | T13 | Prior SBǂ | Fam Hx PET | Smoking |
| Obesity | - | NAǁ |  | *●*  Obesity🡪 T2DM | ●  Obesity🡪 OSA |  | *●*  Obesity🡪  HTN | *●*  Obesity/ overweight🡪  Stage 1 HTN | *Insufficient evidence* |  | NAł | NAǂ | NA | NAǂ | NA | *●*  Smoking 🡪 *reduces* obesity |
| Overweight | NAǁ | - |  | *●*  Overweight🡪 T2DM | ●  Overweight🡪 OSA |  | *●*  Overweight  🡪 HTN |  | *Insufficient evidence* |  | NAł | NAǂ | NA | NAǂ | NA | *●*  Smoking 🡪 *reduces* overweight |
| T2DM | ●  Obesity 🡪 T2DM | *●*  Overweight🡪 T2DM |  | - | *●*  OSA🡪 T2DM |  | ●  DM🡪 cHTN | ●  DM🡪 Stage 1 HTN | *●*  DM🡪 Elevated BP |  | NAł | NAǂ | NA | NAǂ | NA | *●*  Smoking 🡪 T2DM |
| OSA | ●  Obesity🡪 OSA | ●  Overweight🡪 OSA |  | *●*  OSA🡪 T2DM | - |  | -  OSA🡪  cHTN | *Insufficient evidence* | *Insufficient evidence* |  | NA | NAǂ | NA | NAǂ | NA | *●*  Smoking 🡪OSA |
| cHTN | ●  Obesity🡪  HTN | *●*  Overweight  🡪 HTN |  | ●  DM🡪 cHTN | -  OSA🡪  cHTN |  | - | NAǁ | NAǁ |  | NAł | NAǂ | NA | NAǂ | NA | *●*  Smoking 🡪cHTN |
| Booking Stage 1 HTN | *●*  Obesity/ overweight 🡪  Stage 1 HTN | |  | ●  DM🡪 Stage 1 HTN | *Insufficient evidence* |  | NAǁ | - | NAǁ |  | NAł | NAǂ | NA | NAǂ | NA | *Insufficient evidence* |
| Booking ‘elevated BP’ | *Insufficient evidence* | *Insufficient evidence* |  | *●*  DM🡪 Elevated BP | *Insufficient evidence* |  | NAǁ | NAǁ | - |  | NAł | NAǂ | NA | NAǂ | NA | *Insufficient evidence* |
| APAS | NAł | NAł |  | NAł | NA |  | NAł | NAł | NAł |  | - | NAǂ | NA | NAǂ | NA | NA |
| Prior PETǂ | NAǂ | NAǂ |  | NAǂ | NAǂ |  | NAǂ | NAǂ | NAǂ |  | NAǂ | - | NAǂ | NAǂ | NAǂ | NAǂ |
| T13 | NA | NA |  | NA | NA |  | NA | NA | NA |  | NA | NAǂ | - | NAǂ | NA | NA |
| Prior SBǂ | NAǂ | NAǂ |  | NAǂ | NAǂ |  | NAǂ | NAǂ | NAǂ |  | NAǂ | NAǂ | NAǂ | - | NAǂ | NAǂ |
| Fam Hx PET | NA | NA |  | NA | NA |  | NA | NA | NA |  | NA | NAǂ | NA | NAǂ | - | NA |
| Smoking | *●*  Smoking 🡪 *reduces* obesity | *●*  Smoking 🡪 *reduces* overweight |  | *●*  Smoking 🡪 T2DM | *●*  Smoking 🡪OSA |  | *●*  Smoking 🡪cHTN | *Insufficient evidence* | *Insufficient evidence* |  | NA | NAǂ | NA | NAǂ | NA | - |

*NA = not applicable, because the categories are mutually exclusive (e.g., obesity and overweight, or booking ‘elevated BP’ or ‘stage 1 htn’), or the relationship is an impossibility (e.g., medical conditions causing a chromosomal abnormality, which is related either to maternal age or a familial or spontaneous mutation)*

** For strength of association between clinical risk factors, ‘definite’ is represented by red dots (●), ‘probable’ by black dots (●), and ‘possible’ by grey dots (●).* *The direction of a potential causal relationship is stated; whether the relationship could be bidirectional, this is indicated by ‘↔’ and the most likely direction is specified. Quality of evidence is represented by shading: high quality as* ***GREEN****, moderate quality as* ***YELLOW****, low quality as* ***BLUE,*** *very low quality as* ***PURPLE****; and the lack of evidence meeting our criteria is represented by* ***GREY*** *shading.*

*ł Any relationship between APAS and obesity, T2DM, or elevated booking BP would be explained by an intermediate pathway (e.g., renal disease or therapy with steroids).*

*ǂ We have information on the index pregnancy, and have not made any assumption about whether or not a chronic condition was present at the time of the previous pregnancy complication.*

*ǁ These risk factors are highly intercorrelated.*

| **Associations** | **Reference** | **Participants** | **Strength of association** | **Quality of evidence** | **Notes** |
| --- | --- | --- | --- | --- | --- |
| Obesity (BMI ≥ 30) -> T2DM | Field JAMA Internal Med 2001 | 77,690 women from the Nurses' Health Study in the United States | Definite (OR 10.0, 95% CI: 8.4-11.8) | High | For BMI = 30.0–34.9; even higher for ≥35 (OR 17.0, 95% CI: 14.2-20.5) |
| Overweight (BMI = 25.0–29.9) -> T2DM | Field JAMA Internal Med 2001 | 77,690 women from the Nurses' Health Study in the United States | Definite (OR 4.6, 95% CI: 3.9-5.4) | High |  |
| Obesity (BMI ≥ 30) -> OSA | Rice BMC Pregnancy Childbirth 2015 | 1,032 pregnant women (24-28 wks GA) in Lima, Peru | Definite (OR 13.23, 95% CI: 6.25–28.01) | High | Aligns with Peppard 2013 (RR 9.38, Table 2) |
| Overweight (BMI = 25.0–29.9) -> OSA | Rice BMC Pregnancy Childbirth 2015 | 1,032 pregnant women (24-28 wks GA) in Lima, Peru | Definite (OR 3.69, 95% CI: 1.82–7.50) | High | Aligns with Peppard 2013 (RR 2.92, Table 2) |
| Obesity (BMI ≥ 30) -> hypertension | Field JAMA Internal Med 2001 | 77,690 women from the Nurses' Health Study in the United States | Probable (OR 2.1, 95% CI: 1.9-2.2) | High | For BMI = 30.0–34.9; even higher for ≥35 (OR: 2.3, 95% CI: 2.1-2.6)  Dose-response relationship even within normal BMI (Fig 2, Willett NJEM 1999) |
| Overweight (BMI = 25.0–29.9) -> hypertension | Field JAMA Internal Med 2001 | 77,690 women from the Nurses' Health Study in the United States | Probable (OR 1.7, 95% CI: 1.6-1.8) | Moderate | Dose-response relationship even within normal BMI (Fig 2, Willett NJEM 1999) |
| Overweight/ Obesity (BMI ≥25) -> stg 1 hypertension (pregnancy sBP ≥ 130 or dBP ≥ 80 mmHg) | Wu Hypertension 2020 | 47 874 pregnant women with BP <140/90 mm Hg in Shanghai, China | Probable (OR 2.44, 95 % CI: 2.28- 2.62) | Low | Calculated from Table 1  OR = (ad) / (bc)  A) Stage 1 Hypertension outcome in exposed (BMI ≥25) group: 1420  B) Normotensive outcome in exposed (BMI ≥25) group: 2617 C) Stage 1 Hypertension outcome in non-exposed (BMI 18.5–24.9) group: 6878 D) Normotensive outcome in non-exposed (BMI 18.5–24.9) group: 30 990 |
| Overweight/ Obesity (BMI ≥25) -> elevated BP (pregnancy 120–129 mmHg with dBP < 80 mmHg) | N/A | N/A | N/A | N/A | No evidence found for specifically elevated BP, but higher pre-pregnancy BMI associated with higher pregnancy SBP (0.25 mm Hg/kg/m2; 95% CI 0.17 to 0.34; p<0.01) and DBP (0.18 mm Hg/kg/m2; 0.13 to 0.24; p<0.01) (Savirti BMJ Open 2016) |
| Smoking -> *reduces* obesity | Patel Am J Health Behav 2013 | 22,949 Black and 7831 White women from the Southern Community Cohort Study in the United States | Probable (OR 0.42, 95% CI: 0.38 - 0.46 Black women/ OR 0.42, 95% CI: 0.36 - 0.50) | Moderate | For class 1 obesity (30 ≤BMI ≤34.9); lower rates for higher classes of obesity; Supported by risk of weight gain associated with quitting (Bush Obesity 2016, Tian Obesity Reviews 2015) |
| Smoking -> *reduced* overweight (BMI = 25.0–29.9) | Patel Am J Health Behav 2013 | 22,949 Black and 7831 White women from the Southern Community Cohort Study in the United States | Probable (OR 0.59, 95% CI: 0.54 - 0.65 for Black women / OR 0.59, 95% CI: 0.50 - 0.70 for White women) | Moderate | Supported by risk of weight gain associated with quitting (Bush Obesity 2016, Tian Obesity Reviews 2015) |
| OSA -> T2DM | Wang JDI 2022 | 150,030 participants, 8 cohort studies in Korea, Australia, Canada, Taiwan, USA, Finland, Hong Kong and Sweden | Probable (OR 2.15, 95% CI 1.68–2.75) | Moderate | Male and female participants included, but sensitivity analyses showed that there were no significant differences between studies with male ≤ female groups and male > female groups |
| T2DM -> cHTN | Conen BMJ 2007 | 39, 322 women from the Women's Health Study in the United States | Definite (OR 9.90, 95%CI: 7.85-12.48) | Low | Exposure was history of diabetes  Calculated from Table 1  OR = (ad) / (bc)  A) Hypertension outcome in exposed (Diabetes) group: 639 B) Normotensive outcome in exposed (Diabetes) group: 82 C) Hypertension outcome in non-exposed (No diabetes) group: 9812 D) Normotensive outcome in non-exposed (No diabetes) group: 12463  In 2DM, substantial proportion of patients already have hypertension at the time of diabetes diagnosis, so timelines unclear. Statements are made that there is an association, which may be based on a number of factors (CKD, expanded plasma vol, arterial stiffness)  However, findings are supported by Fox et al TCM 2010 & Sun et al Circ Res 2019 (genetically instrumented T2D was associated with risk of HTN (OR 1.07 [95% CI, 1.04–1.10]) while the genetically determined HTN showed no relationship with T2D (OR 0.96 [0.88–1.04]). T2D was associated with a 0.67 mm Hg higher SBP (95% CI 0.41–0.93), but not with a higher DBP (Sun et al 2019). |
| T2DM -> stg 1 hypertension (sBP ≥ 130 or dBP ≥ 80 mmHg) | Conen BMJ 2007 | 39, 322 women from the Women's Health Study in the United States | Definite (OR 4.49, 95%CI: 3.41-5.90) | Low | Exposure was history of diabetes  Calculated from Table 1  OR = (ad) / (bc)  A) Stage 1 Hypertension outcome in exposed (Diabetes) group: 143 B) Normotensive outcome in exposed (Diabetes) group: 82 C) Stage 1 Hypertension outcome in non-exposed (No diabetes) group: 4842 D) Normotensive outcome in non-exposed (No diabetes) group: 12463 |
| T2DM -> elevated BP (120–129 mmHg with dBP < 80 mmHg) | Conen BMJ 2007 | 39,322 women from the Women's Health Study in the United States | Probable (OR 2.08, 95% CI: 1.59-2.73) | Very Low | Exposure was history of diabetes  Calculated from Table 1  OR = (ad) / (bc)  A) Elevated BP outcome in exposed (Diabetes) group: 153 B) Normotensive outcome in exposed (Diabetes) group: 82 C) Elevated BP outcome in non-exposed (No diabetes) group: 11163 D) Normotensive outcome in non-exposed (No diabetes) group: 12463 |
| Smoking -> T2DM | Yuan J Public Health 2019 | 5,077,289 participants from 20 cohort studies in Norway (2 studies), USA (5 studies), Japan, Sweden, Finland, Germany (2 studies), Netherlands, UK, Korea, Japan, and multi-country in Europe | Possible (RR 1.27, 95% CI: 1.19–1.35) | Moderate | Male and female participants included, but separate analysis conducted for women and similar risk of type 2 diabetes associated with smoking in women compared with men (RR: 0.98, 95% confidence interval [CI]: 0.96–1.01) |
| OSA -> hypertension | Cano-Pumarega Chest 2017 | 1155 participants from the Vitoria Sleep Cohort in Spain | Unlikely (not significant for women either hypertension or severe hypertension OR 2.14, 95% CI: 0.40-11.36) | Very Low | OR reported for severe hypertension only (≥160/100mmHg)  Moderate/severe OSA significantly associated with severe hypertension in men (OR 2.54, 95% CI: 1.09-5.95) but not women |
| Smoking -> OSA | Ioannidou Medicina 2021 | 1115 women from Greece | Unlikely (OR 0.96, 95% CI: 0.7–1.3) | Very Low |  |
| Smoking -> hypertension | Bowman J Am Coll Cardiol 2007 | 28,236 women from the Women's Health Study in the United States | Unlikely for current smokers of 1-14 cigarettes a day (HR 1.02, 95% CI 0.92 to 1.13); possible for >15 cigarettes a day (OR 1.11, 95% CI 1.03-1.21) | Moderate |  |

**Table S3:** Indirect relationships for SOCIAL risk factors for pre-eclampsia, that are definite/probable in nature, based on high/moderate quality evidence*

|  | Work stress | Whole body vibrations | Prolonged bending | Lack of ANC | Distance to health facility | Heat  exposure in  early  pregnancy | UV-B exposure (protective) | Calcium and/or vitamin D supplementation (protective) |
| --- | --- | --- | --- | --- | --- | --- | --- | --- |
| Work stress | - | *Insufficient evidence* | *Insufficient evidence* | *●*  Maternal stress 🡪 fewer ANC visits | NA | *●*  Heat exposure 🡪 Maternal stress | *Insufficient evidence* | NA |
| Whole body vibrations | *Insufficient evidence* | - | NA | NA | NA | NA | NA | NA |
| Prolonged bending | *Insufficient evidence* | NA | - | NA | NA | NA | NA | NA |
| Lack of ANC | *●*  Stressful life events 🡪 fewer ANC visits | NA | NA | - | *●*  Distance to health facility 🡪 Delayed ANC | *Insufficient evidence* | *Insufficient evidence* | ●  High ANC attendance 🡪 calcium supplementation |
| Distance to health facility | NA | NA | NA | *●*  Distance to health facility 🡪 Delayed ANC | - | *Insufficient evidence* | *Insufficient evidence* | *Insufficient evidence* |
| Heat  exposure in early pregnancy | *●*  Heat exposure 🡪 Maternal stress | NA | NA | *Insufficient evidence* | *Insufficient evidence* | - | NAǁ | NA |
| UV-B exposure (protective) | *Insufficient evidence* | NA | NA | *Insufficient evidence* | *Insufficient evidence* | NAǁ | - | NA |
| Calcium and/or vitamin D supplementation (protective) | NA | NA | NA | ●  High ANC attendance 🡪 calcium supplementation | *Insufficient evidence* | NA | NA | - |

NA = not applicable, because the categories are mutually exclusive, or the relationship is an impossibility.

* For strength of association between clinical risk factors, ‘definite’ is represented by red dots (●), ‘probable’ by black dots (●), and ‘possible’ by grey dots (●). The direction of a potential causal relationship is stated; whether the relationship could be bidirectional, this is indicated by ‘↔’ and the most likely direction is specified. Quality of evidence is represented by shading: high quality as GREEN, moderate quality as YELLOW, low quality as BLUE, very low quality as PURPLE; and the lack of evidence meeting our criteria is represented by GREY shading.

ǁ These risk factors are highly intercorrelated.

| **Associations** | **Reference** | **Participants** | **Strength of association** | **Quality of evidence** | **Notes** |
| --- | --- | --- | --- | --- | --- |
| Maternal stress 🡪 Fewer ANC visits | Brown BMC Public Health 2011 | 4,366 women from Australia | Probable (OR 2.17, 95% CI:1.4-3.4) | Moderate | Exposure was maternal stress defined as stressful life events (including work related issues) rather than work stress. Outcome was fewer ANC visits (<5 ANC visits vs ≥ 5 visits) rather than absolute lack of ANC;  Dose response: OR 2.17 (1.4-3.4) for ≥3 issues and OR 1.6 (1.1-2.3) for 1-2 issues |
| Heat exposure 🡪 Maternal stress | Lin Environmental Research 2017 | 1931 pregnant women from China | Probable (OR 2.9, 95% CI: 1.9-4.6) | Low | Exposure was high temperatures (95^th^ percentile for study location/period) - 31.2–34.1 ℃. Outcome was maternal emotional stress (highest tertile on the Global Severity Index). |
| Distance to health facility 🡪 Early ANC | Abdo BMC Pregnancy and Childbirth 2023 | 6597 pregnant women, 12 studies from sub-Saharan Africa (Ethiopia, Tanzania and Somalia) | Probable (OR 0.63, 95% CI: 0.41-0.97) | Low | Compared to women who lived far from health facilities, those who lived near health facilities had OR 1.59 (1.03-2.46) for first-trimester ANC contact. We reversed to report far distance as the exposure to fit our conceptual framework. Outcome was delayed ANC rather than absolute lack of ANC. |
| High ANC attendance 🡪 calcium supplementation | Ajong PLOS One 2023 | 1074 pregnant women from Cameroon | Definite (OR 6.01, 95% CI: 3.84–9.34) | High | More ANC visits = more than 3 antenatal care visits.  Findings are reinforced by increased odds of calcium supplements among women with antenatal care onset before 4 months of pregnancy (OR 2.64, 95% CI: 1.84–3.78) |

**Table S4:** Indirect relationships between CLINICAL and SOCIAL risk factors for pre-eclampsia

|  |  | **Clinical determinants** | | | | | | | | | | | | |
| --- | --- | --- | --- | --- | --- | --- | --- | --- | --- | --- | --- | --- | --- | --- |
|  |  | Obesity | Overweight | T2DM | OSA | cHTN | Stage 1 HTN | Elevated BP | APAS | Prior PET | T13 | Prior SB | FAM Hx PET | Smoking |
| **Social determinants** | Work stress | *●*  Work stress 🡪 Higher BMI | | *●*  Work stress 🡪 T2DM | *Insufficient evidence* | -  Work stress 🡪 higher blood pressure | | | NA | NAǂ | NA | NAǂ | NA | *●*  Work stress 🡪 smoking (in pregnancy) |
|  | Occupational whole body vibrations | NA | NA | NA | NA | *Insufficient evidence* | | | NA | NAǂ | NA | NAǂ | NA | NA |
|  | Prolonged bending | NA | NA | NA | NA | *Insufficient evidence* | | | NA | NAǂ | NA | NAǂ | NA | NA |
|  | Lack of ANC | -  Obesity 🡪 Delayed ANC | *●*  Overweight 🡪 Delayed ANC | *Insufficient evidence* | *Insufficient evidence* | *Insufficient evidence* | | | NA | NAǂ | NA | NAǂ | NA | *●*  Smoking 🡪 Fewer ANC visits |
|  | Distance to health facility | NA | NA | NA | NA | *Insufficient evidence* | | | NA | NAǂ | NA | NAǂ | NA | NA |
|  | Heat  exposure in early pregnancy | NA | NA | NA | NA | -  Heat exposure in early pregnancy 🡪 elevated blood pressure or hypertension | | | NA | NAǂ | NA | NAǂ | NA | NA |
|  | UV-B exposure (protective) | NA | NA | NA | NA | *●*  Sun exposure 🡪 HTN | *Insufficient evidence* | *Insufficient evidence* | NA | NAǂ | NA | NAǂ | NA | NA |
|  | Calcium and/or vitamin D supplementation (protective) | NA | NA | NA | NA | *Insufficient evidence* | | | NA | NAǂ | NA | NAǂ | NA | NA |

| **Associations** | **Reference** | **Participants** | **Strength of association** | **Quality of evidence** | **Notes** |
| --- | --- | --- | --- | --- | --- |
| Work stress 🡪 Higher BMI | Roos Arch Environ Occup Health 2013 | 5,786 women from the Helsinki Health Study in Finland | Possible (RR 1.15, 95% CI: 0.95–1.38) | Low | Compared to women with low job strain, women with high job strain did not have significantly higher odds of weight gain  These results align with systematic review of cohort studies on job strain and risk of obesity (Kivimaki et al 2015), that included three studies reporting results for women, though did not do a separate meta-analysis by sex  Fujishiro et al 2015 – study with women from the Nurses’ Health Study II (n=52 656) - women who reported high job strain at least once during the four-year period had a greater increase in BMI (ΔBMI=0.06–0.12, p<0.05) than those who never reported high job strain. The greater the baseline BMI, the greater the BMI gain associated with consistently high job strain |
| Work stress 🡪 T2DM | Sui PLOS One 2016 | 138,310 women across 4 studies | Possible (RR 1.22, 95% CI: 1.01–1.46) | High | Work-related stress measuring job strain specifically; effect was not found in men  Among women, work stress was associated with higher risk of T2DM in the obese (HR 2.01: 1.06; 3.92) but not in the nonobese (P INTERACTION = 0.005) – Heraclides et al 2012 |
| Work stress 🡪 Increased blood pressure | Guimont 2006 | 3236 women from Canada | Unlikely (RR systolic 1.15, 95% CI: 0.93 -1.41/ RR diastolic 1.06, 95% CI: 0.85-1.31) | Very low | Never exposed vs exposed (baseline/follow-up).  Results align with systematic review and a small study from Canada. Gilbert-Ouimet et al 2014 – systematic review of psychosocial work factors and blood pressure - Of the 12 prospective studies on job strain, 5 presented separate results for men and 4 presented separate results for women. In studies on BP level, a higher proportion observed a deleterious effect among men (5/5 studies) compared to women (2/4 studies). Moreover, the only study on hypertension reported an effect among men versus no effect among women  Lavigne-Robichaud et al 2019 - Female (n = 363) white collar workers from Quebec. Job strain not significantly associated with prevalence of uncontrolled hypertension (Prevalence ratio PR 1.14, 95% CI: 0.73–1.78, uncontrolled hypertension defined as ≥135/85 mmHg for non-diabetic participants and ≥125/75 mmHg for diabetic participants). Note that associations were significant in men (PR 1.46, 95% CI: 1.07–1.98, n=354 |
| Work stress 🡪 smoking | Mattson Scand J Work Environ Health 2021 | 65, 645 pregnancies from the Danish National Birth Cohort (1996–2002) | Possible (OR 1.41, 95% CI: 1.08–1.83) | Low | Non-smoking women exposed to high strain work were more likely to become daily smokers compared to non-smoking women exposed to low strain work |
| Obesity 🡪 Late ANC | Barber BMC Pregnancy and Childbirth 2017 | 619,502 births from England | Unlikely (OR 1.04, 95%CI 1.02–1.06) | Low | In comparison to women with normal weight, obese women had higher odds of late antenatal care booking (>13+6 weeks), but the effect size was small. Adjusted for maternal age, ethnic group, parity, Index of Multiple Deprivation score and employment status. |
| Overweight 🡪 Late ANC | Barber BMC Pregnancy and Childbirth 2017 | 619,502 births from England | Possible (OR 1.11, 95%CI 1.09–1.12 | Low | In comparison to women with normal weight, overweight had higher odds of late antenatal care booking (>13+6 weeks). Note that underweight women had reduced odds (aOR 0.77, 95%CI 0.74–0.81) had reduced odds of late antenatal care booking. |
| Smoking 🡪 Fewer ANC visits | Joshi BMC Pregnancy and Childbirth 2014 | 4,079 women from Nepal Demographic and Health Survey 2011 | Possible (aOR 1.40, 95% CI: 1.02-1.92) | Low | Compared to women who smoked, non-smokers had higher odds of four or more antenatal care visits () after adjusting for potential confounders (maternal age, education, work status, wealth quintile, religion, decision-making, media exposure, parity, intended pregnancy, family planning, ecological zone of residence, residence, husband’s education, husband’s occupation) |
| Heat exposure in early pregnancy 🡪 high blood pressure/ hypertension | Part Environmental Research 2022 | 8503 births from South Africa | Unlikely | Very Low | There was a tendency towards an increased risk of hBP with high temperatures during the first few weeks of pregnancy, but no significant associations after adjustment – Fig 1 and Table S3. While no significant association with high blood pressure, the study did find high mean temperatures at 2-5 weeks’ gestation associated with risk of pre-eclampsia. |
| UV-B 🡪 hypertension | Lindqvist Photochemical & Photobiological Sciences 2021 | 23,593 women from the Melanoma in Southern Sweden (MISS) cohort | Possible (OR 1.41, 95% CI 1.3‒1.6) | Moderate | Hypertension was measured by the proxy “use of hypertension medication”  Evidence of a dose-response: Compared to women with the highest sun exposure, those with the lowest had OR 1.41 (95% CI 1.3‒1.6) and moderate had OR 1.15 (95% CI 1.1‒1.2) higher odds of hypertension. |
| Supplementation 🡪 high blood pressure | Cormick Cochrane 2022 | 1915 women in 8 studies | See note |  | Calcium supplementation/fortification vs control. None of the studies reported hypertension as a dichotomous outcome, though there may be an effect on calcium supplementation on diastolic blood pressure in women. The effect on systolic and diastolic blood pressure for women was: MD ‐1.25, 95% CI ‐2.53, 0.03; 1915 participants; eight studies; I^2^ = 0%, high‐certainty evidence; MD ‐1.04, 95% CI ‐1.86, ‐0.22; 1915 participants; eight studies; I^2^ = 4%, high‐certainty evidence, respectively.  Review of the effects of calcium plus vitamin D supplementation also did not find any studies in women that reported hypertension as a dichotomous outcome (Wu et al 2017) |
